# DNA metabarcoding allows non-invasive identification of arthropod prey provisioned to nestling Rufous hummingbirds (*Selasphorus rufus*)

**DOI:** 10.7717/peerj.6596

**Published:** 2019-03-05

**Authors:** Alison J. Moran, Sean W.J. Prosser, Jonathan A. Moran

**Affiliations:** 1 Hummingbird Project, Rocky Point Bird Observatory, Victoria, British Columbia, Canada; 2 Centre for Biodiversity Genomics, Biodiversity Institute of Ontario, University of Guelph, Guelph, Ontario, Canada; 3 School of Environment and Sustainability, Royal Roads University, Victoria, British Columbia, Canada

**Keywords:** eDNA, Arthropod diet, Provisioning, Nestling, Rufous hummingbird, Non-invasive sampling

## Abstract

Hummingbirds consume sugars from nectar, sap and honeydew, and obtain protein, fat and minerals from arthropods. To date, the identity of arthropod taxa in hummingbird diets has been investigated by observation of foraging or examination of alimentary tract contents. Direct examination of nestling provisioning adds the extra complication of disturbance to the young and mother. Here, we show that arthropod food items provisioned to Rufous hummingbird (*Selasphorus rufus*) nestlings can be identified by a safe and non-invasive protocol using next-generation sequencing (NGS) of DNA from nestling fecal pellets collected post-fledging. We found that females on southern Vancouver Island (British Columbia, Canada) provisioned nestlings with a wide range of arthropod taxa. The samples examined contained three Classes, eight Orders, 48 Families, and 87 Genera, with from one to 15 Families being identified in a single pellet. Soft-bodied Dipterans were found most frequently and had the highest relative abundance; hard-bodied prey items were absent from almost all samples. Substantial differences in taxa were found within season and between years, indicating the importance of multi-year sampling when defining a prey spectrum.

## Introduction

Hummingbirds (Trochilidae) are conspicuous nectarivores (see Abrahamczyk & Kessler, 2015 for a recent review) and their sugar metabolism has been investigated in detail (McWhorter et al., 2006); however, they are also predators of small arthropods (e.g., Chavez-Ramirez & Dowd, 1992; Chavez-Ramirez & Tan, 1993; Stiles, 1995). Arthropod prey supply protein and fat, as well as replacing many of the salts lost in clearance through the kidneys (Calder & Hiebert, 1983; Lotz & Martínez Del Rio, 2004).

To date, analysis of food items in birds has been undertaken via administration of emetics (Poulin, Lefebvre & McNeil, 1994a; Poulin, Lefebvre & McNeil, 1994b), collection of bolus from captured birds (Knight et al., 2018), examination of stomach, crop or gizzard contents from dead specimens (Remsen, Stiles & Scott, 1986; McCloskey, Thompson & Ballard, 2009; Powers et al., 2010), or direct observation of birds foraging in the wild (e.g., Young, 1971; Montgomerie & Redsell, 1980; Stiles, 1995; Hayes et al., 2000; Toledo & Moreira, 2008; Powers et al., 2010). While useful, these approaches are generally limited to high taxonomic level assignment (Order, Family), due to problems in identifying digested fragments or the ability of the observer to classify small prey items at distance under field conditions. Stable isotope analysis of hummingbird tissues (*δ*^15^N) has been used to infer trophic level of the invertebrate prey (Hardesty, 2009), but again, fine-scale taxonomic discrimination is limited.

Analysis of provisioning to nestlings produces a further set of complications in that nest disturbance can be a problem. For bird species that use artificial nest boxes, video cameras have been used successfully to analyze prey supplied to nestlings (e.g., Currie, Nour & Adriaensen, 1996; Seress et al., 2017; Tomotani et al., 2017; Sinkovics et al., 2018). However, this approach is not feasible for hummingbirds, as females regurgitate food directly into the crop of nestlings, precluding meaningful quantitative analysis of prey items via direct observation (J Moran, pers. obs., 2017).

Recent advances in DNA analysis have allowed researchers to assign fine-scale taxonomic identity to a range of biological materials. The mitochondrial cytochrome *c* oxidase subunit I (COI) gene has sufficient variability to allow species identification and/or the assignment of a barcode index number (BIN) (Ratnasingham & Hebert, 2013), and substantial effort has gone into building a large taxonomic reference database (BOLD Systems, http://www.boldsystems.org). Since rapid DNA degradation is common in certain sample types, such as fecal pellets, mini-barcodes (i.e., short informative regions of COI) have been developed for investigating dietary composition (e.g., arthropod-specific primer sets; Zeale et al., 2011). Several studies to date have confirmed the utility of fecal material as a source of identifiable DNA from dietary items in insects (Kaunisto et al., 2017), fishes (Corse et al., 2010), mammals (Deagle et al., 2005; Clare et al., 2009; Zeale et al., 2011; Vesterinen et al., 2013; Mallott, Mahli & Garber, 2015; Berry et al., 2017; Sugimoto et al., 2018) and birds (Oehm et al., 2017; Jedlicka, Vo & Almeida, 2017; McInnes et al., 2016; Sullins et al., 2018).

Given these recent advances, we realized that it might be possible to examine the diet of Rufous hummingbird (*Selasphorus rufus* Gmelin) nestlings non-invasively, because they project cloacal fluid, which contains a fecal pellet and dilute urine, up to 20 cm from the nest (J Moran, pers. obs., 2017). Furthermore, the fecal pellets remain intact for a long time after fledging. Typically, adult female Rufous hummingbirds do not defecate around the nest (J Moran, pers. obs., 2017) and so nestlings generate the majority if not all fecal pellets decorating a nest and its surrounding foliage. As cloacal fluid weathers throughout the nestling period (approximately 3 wk), only the hard, black fecal pellets remain for sampling. We tested the use of next-generation sequencing (NGS) of DNA from these pellets as a means of allowing fine-scale taxonomic identification of prey items. Female hummingbirds provision nestlings with a range of arthropod prey that is likely to vary within and between years, but accessing fecal samples while juveniles are still in the nest can be difficult and may cause an inappropriate level of disturbance. The overall aim of this study was to determine whether or not arthropod prey taxa could be identified by amplification of DNA from nestling fecal pellets collected post fledging. We sampled fecal pellets from foliage adjacent to Rufous hummingbird nests active in 2017 and 2018, and amplified mini barcodes using an arthropod-specific primer set (Zeale et al., 2011).

## Materials & Methods

### Nestling fecal pellet sampling

Fieldwork was undertaken in coniferous forests (Coastal Douglas-fir Moist Maritime Subzone) on southern Vancouver Island, British Columbia, Canada (48°25′N, 123°28′W; elevation 40–80 m). We collected fecal pellets adhering to Western Red Cedar (*Thuja plicata*) foliage 1–3 m above ground and adjacent (≤30 cm) to *S. rufus* nests active during 2017 and 2018 (five from each year). The nests were not disturbed and all samples were collected at least one week after both young had successfully fledged; one nest was sampled the year following active use. Since nest reuse is common in Rufous hummingbirds on Vancouver Island (J Moran, pers. obs., 2017), we were able to obtain samples from two nests that were used in both years. We did not collect samples directly from nests, as spider’s webs are used in nest construction (A Moran, pers. obs., 2017; Healy & Calder, 2006), and contamination from web material could potentially result in amplification of DNA from non-prey arachnids. After nesting was completed, the foliage surrounding each nest area was cleared of pellets so no previous years’ samples would be collected in the following year should that nest be reused. We collected pellets directly from the foliage into new Ziploc® bags, which were maintained on ice until transfer to −20 °C for storage (within 5 h). For analysis, single or multiple pellets (≤5) were transferred to 2 ml cryotubes (Fisher Scientific, Waltham, MA, USA) and maintained at −20 °C. Three environmental blanks were obtained from Western Red Cedar foliage >1 m from the ground, at between 5 and 8 m from nests F (Middentop 2018) and J (Middenlow 2018). We applied distilled water to the leaf scales and collected 1.5 ml of wash in a 2 ml cryotube. Following collection, the environmental blank samples were maintained on ice until transfer to −20 °C for storage (within 5 h).

### DNA extraction and PCR amplification

Samples were sent to the Canadian Centre for DNA Barcoding (CCDB, Guelph, ON, Canada) for DNA preparation and analysis. These were suspended in 500 µl Lysis Buffer (700 mM guanidine thiocyanate, 30 mM EDTA pH 8.0, 30 mM Tris-HCl pH 8.0, 0.5% Triton X-100, 5% Tween-20) and 2 mg/ml proteinase K (Promega), homogenized with a Tissue Lyser (Qiagen; 28 Hz, 1 min) and then incubated overnight (18 h) at 56 °C. DNA extraction was then performed using a modified version of the glass fiber technique employed by Ivanova, Dewaard & Hebert (2006) (http://ccdb.ca/resources/). For each sample, 50 µl lysate was mixed with 100 µl Binding Mix (3 M guanidine thiocyanate, 10 mM EDTA pH 8.0, 5 mM Tris-HCl pH 6.4, 2% Triton X-100, and 50% ethanol) and transferred to a silica membrane column (Epoch Biolabs). After centrifugation (6,000 g, 2 min), the membrane was washed once with Protein Wash Buffer (1.56 M guanidine thiocyanate, 5.2 mM EDTA pH 8.0, 2.6 mM Tris- HCl pH 6.4, 1.04% Triton X-100, and 70% ethanol) and twice with 700 µl Wash Buffer (10 mM Tris-HCl pH 7.4, 50 mM NaCl, 0.5 mM EDTA pH 8.0, and 60% ethanol). The membrane was dried by centrifugation (10,000 g, 4 min) followed by incubation at 56 °C for 30 min. DNA was eluted by applying 70 µl Elution Buffer (10 mM Tris-HCl, pH 8) directly to the membrane, allowing it to incubate at room temperature (1 min), and centrifuging at 10,000 g for 5 min. One negative control (Lysis Buffer only) was processed in parallel with the samples.

We employed primers designed to identify arthropods from digested material (Zeale et al., 2011) by amplifying a 157-bp section of the COI barcode region. We used a two-stage fusion primer approach (Prosser & Hebert, 2017) to prepare the amplicons for sequencing on an Ion Torrent PGM sequencing platform (Thermo Fisher, Waltham, MA, USA). Briefly, the first round of amplification contained the COI-specific primers tailed with M13 forward and reverse sequences (ZBJ-ArtF1c_t1, TGTAAAACGACGGCCAAGATATTGGAACWTTATATTTTATTTTTGG and ZBJ-ArtR2_t1, CAGGAAACAGCTATGAWACTAATCAATTWCCAAATCCTCC, respectively; Zeale et al., 2011), and utilized the fecal pellet DNA extracts as template. For a detailed composition of the amplification reaction, see Prosser & Hebert (2017) or http://ccdb.ca/site/wp-content/uploads/2016/09/CCDB_Amplification.pdf. Thermocycling consisted of an initial denaturation for 2 min at 95 °C, followed by 60 cycles (95 °C, 40 s; 53 °C, 40 s; and 72 °C, 30 s), and a final extension of 72 °C for 2 min.

The first-round amplification products were diluted with water (1:1 v:v) and used as template for a second round of amplification. The second round contained fusion primers composed of M13 forward and reverse sequences tailed with Ion Torrent sequencing adapters (Xpress A and Xpress trP1, respectively) and 96 IonXpress multiplex identifier (MID) tags (forward primers only; Thermo Fisher). The final composition of the second-round amplification reactions was the same as the first. Thermocycling consisted of an initial denaturation for 2 min at 95 °C, followed by 5 cycles (95 °C, 40 s; 45 °C, 40 s; and 72 °C, 30 s), 35 cycles (95 °C, 40 s; 51 °C, 40 s; and 72 °C, 30 s), and a final extension of 72 °C for 2 min.

Following the second round of amplification, all samples were pooled, then purified using carboxylate modified magnetic beads (Aline Biosciences, Woburn, MA, USA). DNA fragments >600 bp were removed by mixing 400 µl pooled amplicons with 200 µl magnetic beads, followed by vortexing and incubation at room temperature for 8 min. Beads were collected using a magnetic rack and 550 µl of the supernatant was transferred to a clean tube. Amplicons >100 bp were then selected for by mixing the supernatant with 113 µl dH_2_O and 417 µl magnetic beads. Following a second incubation and pelleting, the supernatant was removed and the beads were washed three times with 1.5 ml 80% ethanol. Beads were air dried for 6 min and then resuspended in 200 µl dH_2_O. The beads were pelleted and 180 µl purified amplicons (i.e., the supernatant) were transferred to a clean tube. The purified DNA was quantified using a Qubit 2.0 fluorometer dsDNA High Sensitivity kit and adjusted to 1 ng/µl.

### Sequencing and bioinformatics

For sequencing on an Ion Torrent PGM, 1,245 µl dH_2_O was added to 5 µl of the 1 ng/µl purified product and then sequenced using a 318 v.2 chip according to the manufacturer’s instructions. The PGM Torrent Browser (Thermo Fisher, Waltham, MA, USA) automatically assigned the resulting reads to samples, using the unique MID tags. The demultiplexed data sets (one for each sample and negative control) were processed through a bioinformatic pipeline (Prosser & Hebert, 2017). Briefly, reads <100 bp or with QV < 20 were removed, and primers and adapter sequences were excised. The reads were then de-replicated and clustered into operational taxonomic units (OTUs) with 98% identity. To remove low abundance artefactual reads and chimeric reads (i.e., de-noise the data), only OTUs composed of 10 or more reads were retained (Clarke et al., 2017; Deiner et al., 2017; Alberdi et al., 2018; Serrana et al., 2018). Taxonomy was assigned to OTUs using a BLAST search of a custom reference database composed of unique BINs from the Barcode of Life Data System v4 (BOLD;  http://www.boldsystems.org/index.php).

To further de-noise the data and ensure that only confident results were retained, a custom *R* script was used to filter the BLAST data to remove sequences with <95% identity to a reference sequence. It also removed sequences in which the region of complementarity was <100 bp. Within each sample, OTUs matching to a common reference sequence were combined and their component reads added together to provide a total read count. 

In addition to taxonomic identification, we also assigned prey items to functional groups (or guilds, e.g., predator, herbivore, etc.). This was undertaken starting with the BOLD data portal (http://www.boldsystems.org/index.php/IDS_OpenIdEngine), and following links to further taxonomic resources. With 87 Arthropod Genera, a wide variety of resources was used to help assign a given Genus to a functional group, including taxon-specific websites (e.g.,  http://www.nadsdiptera.org/Tach/AboutTachs/TachOverview.html for Tachinidae; http://chirokey.skullisland.info/ for Chironimidae) and original peer-reviewed literature. An important caveat is that, at the level of Genus (rather than Species), it is impossible in many cases to find sufficient published information to allow assignment to a particular functional group. In addition, large Genera can encompass a correspondingly wide variety of functional groups. As a result, many Genera were assigned Unknown status with regard to functional group (Table S1). It is also important to note that, especially for many Dipterans, adult and larval stages occupy different functional groups. For example, in the Family Tachinidae (e.g., *Phytomyptera* sp., *Trichophora* sp.) adults feed on nectar and pollen, while the larvae are parasitoids (http://www.nadsdiptera.org/Tach/AboutTachs/TachOverview.html). In the current study, we focused on the adult stage only.

### Statistical analysis

We compared similarity of prey composition between nests at the Genus and Family levels via the Chao-Jaccard Similarity Index (Chao et al., 2005), using the EstimateS v. 9.1.0 software package (Colwell, 2016). Due to the problems outlined above in assigning many Genera to functional groups (see Table S1), similarity analyses were not undertaken for these groupings.

### Data availability

All results are presented in Data S1 and S2. Raw sequencing data (i.e., demultiplexed FASTQ files directly from the Torrent Browser) and final read sets (i.e., post-bioinformatic processing) for each sample are available at the Sequence Read Archive (https://www.ncbi.nlm.nih.gov/sra) with the accession number PRJNA508770. All software components of the bioinformatic pipeline are publicly available from the sources cited in Prosser & Hebert (2017), and the custom *R* and Python scripts are presented in Data S3 and S4.

## Results

Eighty-seven percent (26/30) of the individual fecal pellets yielded amplifiable DNA sequences, compared to only 70% (7/10) of the multiple-pellet samples. A complete breakdown of the arthropod prey taxa detected in each nest is presented in Data S1 and S2. Of the three environmental blank samples, two produced no amplifiable DNA. The third produced a low number of reads (104) for a cranefly Genus, *Limonia* (Diptera; Limoniidae). A possible source is directly from cranefly fecal matter on the branch. No amplification was obtained from the negative control (Lysis Buffer).

### Relative abundance of prey taxa

**Figure 1:**
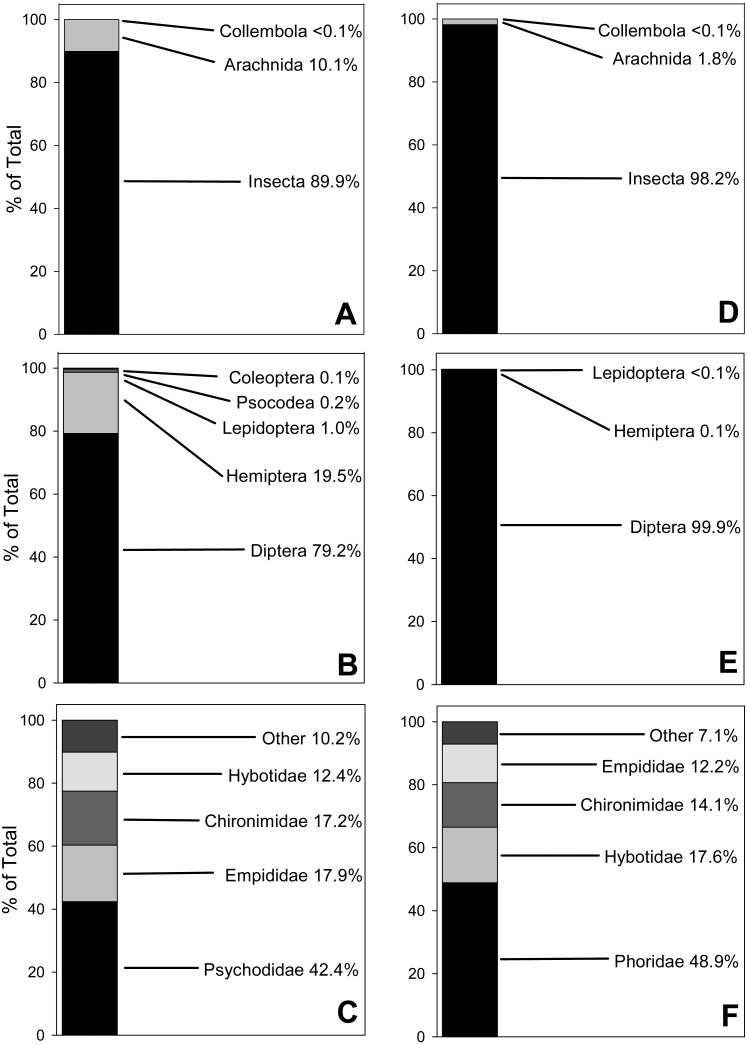
100% Stacked bar charts for relative abundance of prey taxa from 10 *Selasphorus rufus* nests on Southern Vancouver Island, British Columbia, Canada. (A) 2017 Arthropod Classes. (B) 2017 Insect Orders. (C) 2017 Dipteran Families. (D) 2018 Arthropod Classes. (E) 2018 Insect Orders. (F) 2018 Dipteran Families. Relative abundance of each taxon is represented by reads of OTUs. Forty fecal pellet samples in total.

Fecal pellets from 2017 yielded three Classes of prey items (Arachnida [spiders, mites, etc.], Insecta [insects] and Collembola [springtails]), comprising seven Orders, 37 Families and 65 Genera (Table S2). Results for relative (not absolute) abundance, represented by number of reads, showed that Insecta was the most abundantly amplified Class (89.9% of total reads), followed by Arachnida (10.1%) and Collembola (<0.1%; Fig. 1A). At the Ordinal level for insects, true flies (Diptera) accounted for 79.2% of total reads, followed by true bugs (Hemiptera, 19.5%), moths (Lepidoptera, 1%), bark lice (Psocodea 0.2%) and beetles (0.1%; Fig. 1B). The most abundant Dipteran Families were moth flies (Psychodidae, 42.4% of total reads), followed by dagger flies (Empididae, 17.9%), non-biting midges (Chironimidae, 17.2%) and dance flies (Hybotidae, 12.4%; Fig. 1C).

The 2018 nests yielded the same three Classes as the previous year, with Insecta again the most relatively abundant (98.2% of reads), followed by Arachnida (1.8%) and Collembola (<0.1%; Fig. 1D). However, the dataset was depauperate relative to 2017 in terms of lower-order taxa, with only five Orders, 28 Families and 35 Genera (Table S3). At the Ordinal level, true flies (Diptera) accounted for almost all of the reads (99.9%); true bugs (Hemiptera) and moths (Lepidoptera) were much less relatively abundant, at 0.1% and <0.1%, respectively (Fig. 1E). Within the Diptera, humpbacked flies (Phoridae) accounted for 48.9% of reads, followed by dance flies (Hybotidae, 17.6%), non-biting midges (Chironimidae, 14.1%) and dagger flies (Empididae, 12.2%; Fig. 1F). Soft-bodied arthropods were overwhelmingly the most relatively abundant prey type in both years.

**Figure 2:**
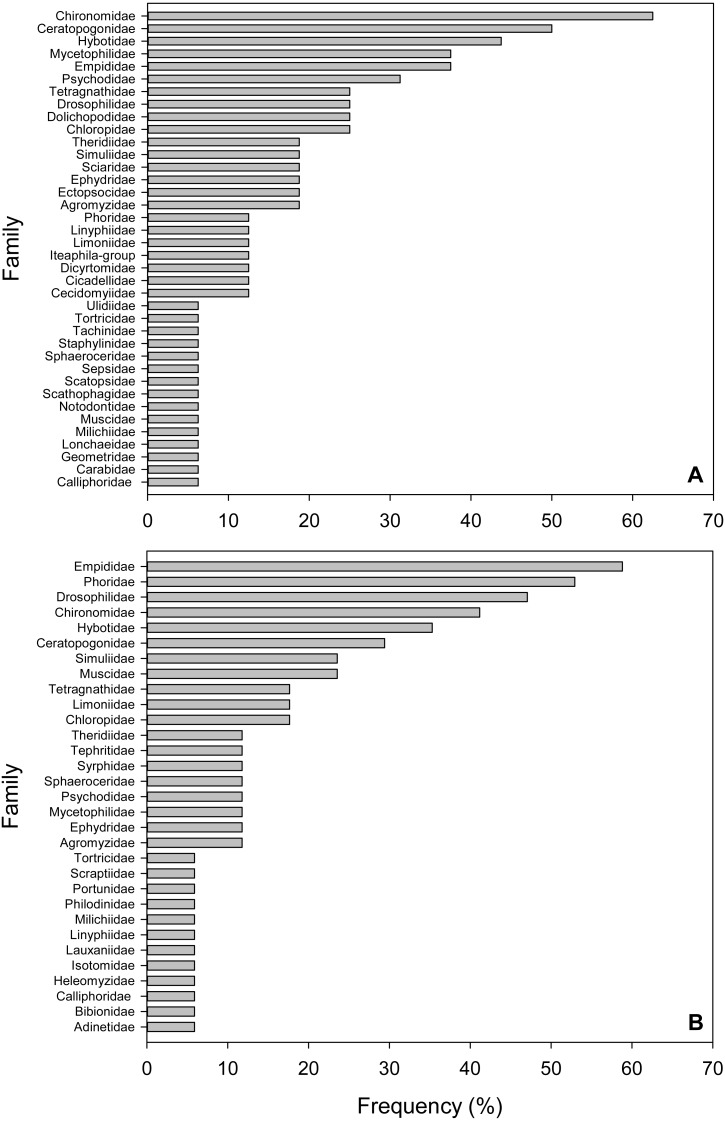
Frequency of Arthropod Families in fecal pellet samples from *Selasphorus rufus* nests on Southern Vancouver Island, British Columbia, Canada. (A) 2017. (B) 2018. Frequency is defined as the percentage of all fecal pellet samples in which a given Family was detected. Five nests per year; four fecal pellet samples per nest.

### Frequencies of arthropod families

We defined frequency of a given taxon as the percentage of total samples in which it was detected. The most frequent Families in the 2017 samples were non-biting midges (Chironimidae), detected in 62.5% of samples, followed by biting midges (Ceratopogonidae, 50%), dance flies (Hybotidae, 43.8%), fungus gnats (Mycetophilidae, 37.5%), dagger flies (Empididae, 37.5%) and moth flies (Psychodidae, 31.3%; Fig. 2A). For the 2018 collection, dagger flies (Empididae) were the most frequent Family (58.8%), followed by humpbacked flies (Phoridae, 52.9%), fruit flies (Drosophilidae, 47%), non-biting midges (Chironimidae, 41.2%), dance flies (Hybotidae, 35.3%) and biting midges (Ceratopogonidae, 29.4%; Fig. 2B). Three Dipteran Families occurred in the top five most frequent lists in both years (Chironimidae, Hybotidae and Empididae). Similar to the relative abundance results above, soft-bodied arthropods were dominant in terms of frequency.

**Figure 3:**
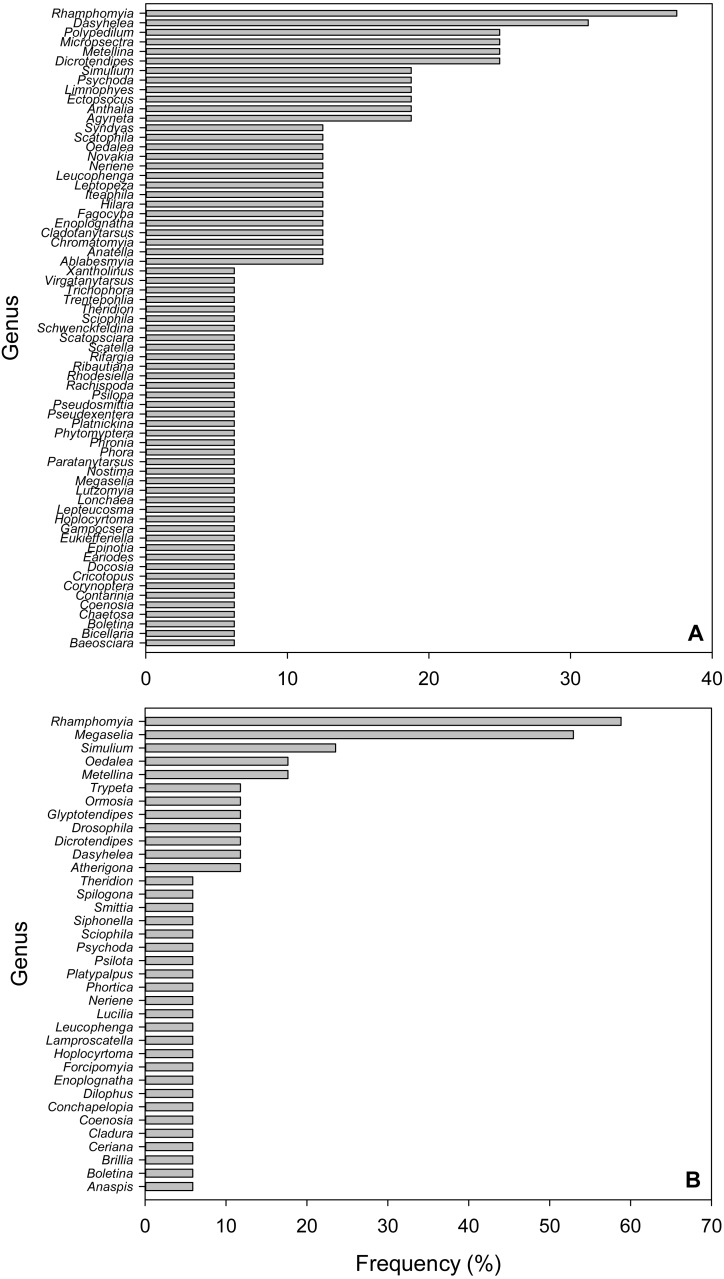
Frequency of Arthropod Genera in fecal pellet samples from *Selasphorus rufus* nests on Southern Vancouver Island, British Columbia, Canada. (A) 2017. (B) 2018. Frequency is defined as the percentage of all fecal pellet samples in which a given Genus was detected. Five nests per year; four fecal pellet samples per nest.

### Frequencies of arthropod genera

Due to the short length (157 bp) of the amplicons used in this study, we only considered taxonomic identifications reliable to Genus. In the 2017 dataset, the most frequent Genus was *Rhamphomyia* (Diptera; Empididae), detected in 37.5% of samples, followed by *Dasyhelea* (Diptera; Ceratopogonidae, 31.3%), *Polypedilum* (Diptera; Chironimidae, 25%), *Micropsectra* (Diptera; Chironimidae, 25%), *Dicrotendipes* (Diptera; Chironomidae, 25%) and the Long-jawed Orb-web spider Genus *Metellina* (Arachnida; Tetragnathidae, 25%; Fig. 3A). In 2018, *Rhamphomyia* (Diptera; Empididae) was again the most frequent Genus (58.8%), followed by *Megaselia* (Diptera; Phoridae, 52.9%), *Simulium* (Diptera; Simuliidae, 23.9%), *Oedalea* (Diptera; Hybotidae, 17.6%) and the Long-jawed Orb-web spider Genus *Metellina* (Arachnida; Tetragnathidae, 17.6%; Fig. 3B).

### Frequencies of functional groups

The prey items included a wide range of functional groups (or guilds): Predators, blood feeders, nectar/pollen feeders, herbivores, sap feeders, fungivores, detritivores, and saprophages (Table S1). Predators were the most frequent group overall, being detected in 81.3% and 88.2% of nests in 2017 and 2018, respectively. Nectar/pollen feeders were the next most frequent group (81.3% and 64.7%). The third most frequent category was Unknown, a result of the difficulty in assigning many Genera to functional group. Blood feeders (which included taxa that feed on arthropod haemolymph) were present in 25.0% and 29.4% of the 2017 and 2018 samples, respectively. Not all functional groups were present in both years. For example, detritivores, sap feeders and saprophages were detected in the 2017 samples, but not in those from 2018 (Table S1).

### Inter-nest similarity

For Families, similarity of prey composition between nests, expressed by Chao’s Abundance-Based-Jaccard Index (Chao et al., 2005), varied from 0.850 (highly similar) to 0.025 (negligible similarity) (Table 1). For Genera, values varied from 0.788 to zero (Table 2). Chao-Jaccard indices overall were higher for Family (mean ± 1 S.E. = 0.357 ± 0.038) than for Genus (0.163 ± 0.028); however, there was no discernable spatial or temporal pattern in the results within each taxonomic level.

Overall, our results show that in temperate regions it is possible to amplify and identify arthropod DNA from hummingbird nestling fecal pellets collected non-invasively >1 wk post-fledging. Whether or not the pellets will remain sufficiently intact for such analysis when collected in the tropics, where the majority of Trochilidae are found, remains to be investigated. Amplification was not successful for all samples; however, failures occurred more often in the larger bulk samples, suggesting either inhibitory effects from the fecal material itself or that excessive amounts of template were present. Typically, high throughput facilities do not normalize DNA concentrations or use extraction procedures unless required. The inhibitory effects observed in large volume samples may be overcome by taking measures such as homogenization and sub-sampling, more stringent DNA purification, or serial dilution of DNA.

**Table 1:** Comparison of arthropod prey similarity at the level of Family between Rufous hummingbird (*Selasphorus rufus*) nests (*n* = 10) on southern Vancouver Island, British Columbia, Canada, in 2017 and 2018. A Chao-Jaccard Estimate value of 1 indicates perfect overlap of prey composition between a pair of nests; a value of zero indicates zero similarity.

**Nest**			**A**	**B**	**C**	**D**	**E**	**F**	**G**	**H**	**I**	**J**
	**Year**		**2017**	**2017**	**2017**	**2017**	**2017**	**2018**	**2018**	**2018**	**2018**	**2018**
			Chartrail	Lagoon1	Serpentine1	Dougcliff	Quarryold	Middentop	Serpentine2	Quarry	Lagoon2	Middenlow
**A**	**2017**	Chartrail	–									
**B**	**2017**	Lagoon1	0.35	–								
**C**	**2017**	Serpentine1	0.18	<0.1	–							
**D**	**2017**	Dougcliff	0.17	0.66	0.19	–						
**E**	**2017**	Quarryold	0.39	0.76	<0.1	0.29	–					
**F**	**2018**	Middentop	0.36	0.73	0.20	0.27	0.74	–				
**G**	**2018**	Serpentine2	0.36	0.29	0.18	0.14	0.12	0.79	–			
**H**	**2018**	Quarry	0.42	0.25	0.13	<0.1	0.25	0.75	0.74	–		
**I**	**2018**	Lagoon2	0.27	<0.1	0.57	<0.1	<0.1	0.21	0.45	0.29	–	
**J**	**2018**	Middenlow	0.27	0.34	0.23	0.8	0.85	0.29	0.74	0.71	<0.1	–

**Table 2:** Comparison of arthropod prey similarity at the level of Genus between Rufous hummingbird (*Selasphorus rufus*) nests (*n* = 10) on southern Vancouver Island, British Columbia, Canada, in 2017 and 2018. A Chao-Jaccard Estimate value of 1 indicates perfect overlap of prey composition between a pair of nests; a value of zero indicates zero similarity.

**Nest**			**A**	**B**	**C**	**D**	**E**	**F**	**G**	**H**	**I**	**J**
	**Year**		**2017**	**2017**	**2017**	**2017**	**2017**	**2018**	**2018**	**2018**	**2018**	**2018**
			Chartrail	Lagoon1	Serpentine1	Dougcliff	Quarryold	Middentop	Serpentine2	Quarry	Lagoon2	Middenlow
**A**	**2017**	Chartrail	–									
**B**	**2017**	Lagoon1	<0.1	–								
**C**	**2017**	Serpentine1	<0.1	<0.1	–							
**D**	**2017**	Dougcliff	<0.1	0.71	<0.1	–						
**E**	**2017**	Quarryold	0.10	0.79	<0.1	0.27	–					
**F**	**2018**	Middentop	<0.1	0.38	<0.1	0.25	0.44	–				
**G**	**2018**	Serpentine2	<0.1	0.27	0.16	0.10	0.13	0.40	–			
**H**	**2018**	Quarry	0.10	<0.1	<0.1	<0.1	0.25	0.45	0.65	–		
**I**	**2018**	Lagoon2	<0.1	<0.1	<0.1	0.11	<0.1	<0.1	0.22	0.04	–	
**J**	**2018**	Middenlow	0.10	0.13	<0.1	0.10	0.13	0.10	0.13	0.13	0.03	–

## Discussion

Hummingbirds spend a considerable part of the day foraging for arthropods and these can represent a vital resource for protein, fatty acids, minerals and vitamins (Gass & Montgomerie, 1981; Stiles, 1995; Powers et al., 2010). Arthropods can also represent an important energetic resource when nectar availability is reduced through seasonal differences in flowering or competition (Wolf, 1970; Hainsworth, 1977; Chavez-Ramirez & Dowd, 1992; Powers et al., 2010). Arthropods are particularly important for juvenile growth, and females provisioning nestlings and newly-fledged young will engage in more arthropod foraging activity than sympatric conspecific males (Stiles, 1995; Wolf, 2008). However, prior to the current study, little was known at a fine-scale taxonomic level about the arthropod taxa provisioned to hummingbird nestlings.

Female Rufous hummingbirds provision young with a wide range of arthropod taxa. Our samples encompassed three Classes, eight Orders, 48 Families, and 87 Genera, with single pellets containing reads from one to 15 Families. Soft-bodied items were overwhelmingly the most frequent and relatively abundant category. Rufous hummingbirds will feed while perching, glean from foliage and are commonly observed hawking (i.e., aerial capture of small prey) from ephemeral, but high-density swarms of small Dipterans, which may account for a combination of limited range of taxa and a high number of reads in any given pellet. With regards to hawking, it is interesting to note that hummingbirds have evolved a rapid jaw-snapping mechanism for catching fast-moving aerial insect prey on the wing (Smith, Yanega & Ruina, 2011).

In our study, Diptera were numerically the dominant taxon in the fecal pellets of nestling Rufous hummingbirds. Some authors have found amplification bias with the ZBJ primers, with higher efficiency achieved for Diptera and Lepidoptera (Clarke et al., 2014) and decreased success for the Orthoptera (Alberdi et al., 2018). However, since we expected Diptera to be the numerically important Order, we chose the ZBJ primers because they were anticipated to provide a large degree of discrimination for that Order and these primers are known to amplify most of the two classes most likely to be found in the hummingbird diet, i.e., Insecta and Arachnida (Zeale et al., 2011; Jedlicka, Vo & Almeida, 2017). Furthermore, a recent study in bats showed correlation between morphological and metabarcoding analyses with elevated detection of Diptera by the latter technique (Hope et al., 2014). Thus, in the case of hummingbird nestlings, metabarcoding of fecal pellets provides a level of discrimination that would be impossible to attain from morphological analyses, given the rapid digestion of small and in most cases, soft-bodied prey items such as small Diptera. In terms of functional groups, a large proportion of the arthropod prey provisioned to the nestlings were predators in adult form (Table S1). Insects that feed on nectar or pollen were also well represented and it is possible that these were gleaned opportunistically on or around flowers visited by the hummingbird while collecting nectar.

A limitation of our approach is that it represents a temporally-integrated picture: since all pellets were collected after fledging, the stage at which a particular pellet was produced (e.g., from a newly-hatched chick or one preparing to fledge), is unknown. In some birds, the variety and quality of prey provisioned to nestlings differs according to habitat quality, prey availability and developmental stage of the young (Orłowski, Frankiewicz & Karg, 2017; Jedlicka, Vo & Almeida, 2017). Our methodology does not provide information on possible changes in the type of prey provisioned to nestlings at different stages of development. Also, we cannot differentiate between pellets produced by individual nestlings. While it may be possible to collect pellets on a daily basis during nestling development, we believe that this approach would be unethical due to the level of stress that such disturbance would cause the female and nestlings. Human activity in close proximity to a nest raises the potential for nest abandonment, early fledging and the alerting of predators and humans to the presence of a nest. Disturbance of a migratory bird nest is illegal in many countries and legal protection for breeding Rufous hummingbirds is provided at the Federal level throughout their breeding range in Canada and the United States. Thus, collection of fecal material before fledging would be problematic from both ethical and legal standpoints.

While recognizing that amplification biases exist (Clarke et al., 2014; Deiner et al., 2017) and that some additional bias might be introduced by different levels of degradation among the samples, we employed number of reads as a very approximate measure of relative (not absolute) abundance within a given taxon, in order to examine how prey taxa and diversity might differ between nest, season and year. Small, soft-bodied Dipterans appeared to be the most relatively abundant and frequent taxon in both 2017 and 2018. However, their dietary importance clearly differs from year to year, as they represented a far greater proportion of the diet in 2018 (98.2% of diet was Insecta, of which 99.9% were Diptera) than in 2017 (89.9% of diet was Insecta, of which 79.2% were Diptera; Fig. 1).

Arachnida have been identified in the gut contents of many species of hummingbirds (Stiles, 1995; Powers et al., 2010). In the current study, they were present in 25% and 17.6% of samples in 2017 and 2018, respectively. However, like Dipterans, their relative abundance in diet differed considerably between the years (10.1% versus 1.8%, in 2017 and 2018, respectively; Fig. 1), possibly reflecting prey availability. Most abundant in both years was *Metellina*, a widespread genus of Long-jawed Orb-web spider. Adult females would be too large for capture by female *S. rufus*, but the smaller males, as well as spiderlings of both sexes, would not. Certainly, the Arachnid content of some tropical hummingbird diets is predominantly composed of web-building spiders (Stiles, 1995) and such spiders may represent an important part of the diet as they have proportionately higher levels of fat and protein than Diptera (Robel et al., 1995; Stiles, 1995). An alternative source of DNA from spiders is via the bird gleaning prey from webs, as observed by Stiles (1995). A third possibility is contamination of the prey item or pellet from the nest itself, as *S. rufus* use spider silk as an adhesive component of the nests (A Moran, pers. obs., 2017). However, we believe this to be unlikely, as females regurgitate prey items directly into the crop of nestlings and the young raise their cloaca clear of the nest prior to ejecting the fecal pellet (A Moran, pers. obs., 2017). All pellets used in the current study were collected from vegetation adjacent to, and not from, nests.

Similar to the wide variety of dietary prey items identified by Stiles (1995), our analyses detected Lepidoptera, Hemiptera, Psocoptera, and Coleoptera as minor dietary components. With respect to the hard-bodied Coleoptera (Staphylinidae, Scraptiidae and Carabidae), identifications were made in only one pellet each. Thus, to paraphrase JBS Haldane’s famous quip, Rufous hummingbird mothers appear to demonstrate an “Inordinate indifference to beetles”, despite some beetles of appropriate size being easily observable in the environment (J Moran, pers. obs., 2017).

In contrast to the results of studies in Costa Rica and Arizona that focussed on adult hummingbirds (Stiles, 1995; Powers et al., 2010), in our study, Hymenoptera were absent as a dietary component. Whether this dietary difference is associated specifically with Rufous hummingbird provisioning remains to be tested, and it may be that the adult females are avoiding wasps and other hard-bodied prey when foraging to feed nestlings. As in the case of beetles, a baseline for relative abundance of potential prey taxa would be required to test this hypothesis with sampling of the foraging habitat for invertebrates (Toledo & Moreira, 2008).

The results of similarity analyses shed some light on the foraging behaviour of individual females. For example, Nests F and J (Middentop 2018 and Middenlow 2018) were used by the same female in 2018 (she was observed being active at and moving between both nests; J Moran, pers. obs., 2018), and are <50 m apart. Nestlings in Nest F (Middentop 2018) were provisioned in May 2018, while those in Nest J (Middenlow 2018) were provisioned in June 2018. Despite proximity and the fact that the same female was provisioning both nests, the Chao-Jaccard Index between them was 0.10, which demonstrates low similarity of prey items at the level of Genus (Table 2). This may show the effect of within-year seasonality on the availability of different prey taxa.

Nest reuse also allowed comparison of between-year similarity. Nest I (Lagoon2 2018) was built on top of the remains of Nest B (Lagoon1 2017), possibly by the same female. Despite the fact that the foraging area would have been similar, the Chao-Jaccard Index for the two was <0.01, demonstrating very low similarity of Genera between years (Table 2). Finally, an analogous situation occurred between Nest C (Serpentine1 2017) and Nest G (Serpentine2 2018), the latter of which was built on the remains of the former. Once again, a low Chao-Jaccard Index of 0.16 indicated low similarity in Genera between years (Table 2). These between-year results (differences between 2017 and 2018 taxa) show the importance of multi-year sampling in defining the prey spectrum of a given hummingbird species.

An unexpected finding was the presence of non-prey sequences in two of the 2018 samples. DNA sequences from two Genera of rotifer were found in Nest H (Quarry 2018); a possible route was via ingestion of a recently-eclosed dipteran that had fed on the rotifers in the larval stage. A potential candidate is the dagger fly *Rhamphomyia* sp., sequences of which were found in the same pellet. Crab DNA was also identified in feces from Nest F (Middentop 2018), which was close to the ocean (<500 m). A potential route was via ingestion of a Dipteran that had fed on a crab carcass (sequences from seven Dipteran Genera were detected in the same pellet). The presence of non-prey DNA raises the possibility that some DNA identified from fecal material may have been transferred to nestlings in the digestive tract of predatory prey items such as spiders, and not from prey items caught directly by the hummingbird.

## Conclusions

The results of this study demonstrate the feasibility of using DNA metabarcoding to identify Arthropod taxa in hummingbird fecal material. Given the importance of hummingbirds as pollinators, a logical next step would be to expand the analysis to include plant DNA in fecal material, in order to identify which species are visited by the bird. Although in the current project the focus was on prey items provisioned to nestlings, the technique will open the door to future research into many other aspects of hummingbird ecology such as niche partitioning between sympatric species, and potential differences in the quality and type of prey items in the diets of adults, juveniles and nestlings. These fundamental ecological questions could be addressed by metabarcoding analysis of the cloacal fluid that is copiously provided by hummingbirds during routine banding.

##  Supplemental Information

10.7717/peerj.6596/supp-1 Data S1 Raw data (Family, Genus, Functional Group) of provisioned invertebrate prey taxa in 10 Selasphorus rufus nests from southern Vancouver Island, British Columbia, Canada

10.7717/peerj.6596/supp-2 Data S2 Taxonomic breakdown (Family, Genus) by nest, of provisioned invertebrate prey taxa in 10 Selasphorus rufus nests from southern Vancouver Island, British Columbia, Canada

10.7717/peerj.6596/supp-3 Data S3 R Script for bioinformatics pipeline

10.7717/peerj.6596/supp-4 Data S4 Python Script for bioinformatics pipeline

10.7717/peerj.6596/supp-5 Table S1 Frequency of arthropod prey by adult functional group in Rufous hummingbird (*Selasphorus rufus*) nests (*n* = 10) on southern Vancouver Island, British Columbia, Canada, in 2017 and 2018 Numbers after taxon are reads.

10.7717/peerj.6596/supp-6 Table S2 Taxonomic breakdown of invertebrates identified from DNA in fecal pellets from five Rufous hummingbird (*Selasphorus rufus*) nests from southern Vancouver Island, British Columbia, Canada, in 2017 Numbers after taxon are reads.

10.7717/peerj.6596/supp-7 Table S3 Taxonomic breakdown of invertebrates identified from DNA in fecal pellets from five Rufous hummingbird (*Selasphorus rufus*) nests from southern Vancouver Island, British Columbia, Canada, in 2018 Numbers after taxon are reads.

## Additional Information and Declarations

### Data Availability

The following information was supplied regarding data availability: BioProject: DNA Metabarcoding allows non-invasive identification of arthropod prey provisioned to nestling Rufous hummingbirds (*Selasphorus rufus*), PRJNA508770.
