# Supplementary material for: DNA metabarcoding allows non-invasive identification of arthropod prey provisioned to nestling Rufous hummingbirds (Selasphorus rufus)
Source: PeerJ. 2019 Mar 5;7:e6596. doi: 10.7717/peerj.6596 (PMC6407503; doi:10.7717/peerj.6596)
Supplement: Table S1 — Numbers after taxon are reads. [file peerj-07-6596-s005.docx]

**Supplemental Table S1:**

**Frequency of arthropod prey by adult functional group in Rufous hummingbird (*Selasphorus rufus*) nests (*n*=10) on southern Vancouver Island, British Columbia, Canada, in 2017 and 2018**.

| **Adult Functional Group** | **Frequency (%) in**  **2017 samples** | **Frequency (%) in**  **2018 samples** |
| --- | --- | --- |
| Predator | 81.3 | 88.2 |
| Nectar/pollen feeder | 81.3 | 64.7 |
| Unknown | 43.8 | 64.7 |
| Blood feeder | 25.0 | 29.4 |
| Detritivore | 18.8 | 0 |
| Fungivore | 12.5 | 5.9 |
| Herbivore | 12.5 | 11.8 |
| Sap feeder | 12.5 | 0 |
| Saprophage | 6.3 | 0 |
