# Supplementary material for: DNA metabarcoding allows non-invasive identification of arthropod prey provisioned to nestling Rufous hummingbirds (Selasphorus rufus)
Source: PeerJ. 2019 Mar 5;7:e6596. doi: 10.7717/peerj.6596 (PMC6407503; doi:10.7717/peerj.6596)
Supplement: Table S2 — Numbers after taxon are reads. [file peerj-07-6596-s006.docx]

**Supplemental Table S2. Taxonomic breakdown of invertebrates identified from DNA in fecal pellets from five Rufous hummingbird (*Selasphorus rufus*) nests from southern Vancouver Island, British Columbia, Canada, in 2017.** Numbers after taxon are reads.

| **Class (*n*=3)** | **Order (*n*=7)** | **Family (*n*=37)** | **Genus (*n*=65)** |
| --- | --- | --- | --- |
| Arachnida 27516 | Araneae 27516 | Linyphiidae 15085 | *Agyneta* 723 |
|  |  |  | *Neriene* 14362 |
|  |  | Tetragnathidae 12177 | *Metellina* 12177 |
|  |  | Theridiidae 254 | *Enoplognatha* 27 |
|  |  |  | *Platnickina* 50 |
|  |  |  | *Theridion* 177 |
| Collembola 120 | Symphypleona 120 | Dicyrtomidae 120 | *-* |
| Insecta 245604 | Coleoptera 254 | Carabidae 11 | *-* |
|  |  | Staphylinidae 243 | *Xantholinus* 243 |
|  | Diptera 194616 | Agromyzidae 1325 | *Chromatomyia* 1231 |
|  |  | Calliphoridae 53 | *-* |
|  |  | Cecidomyiidae 562 | *Contarinia* 11 |
|  |  | Ceratopogonidae 4877 | *Dasyhelea* 3809 |
|  |  | Chironomidae 30181 | *Ablabesmyia* 4295 |
|  |  |  | *Cladotanytarsus* 367 |
|  |  |  | *Cricotopus* 46 |
|  |  |  | *Dicrotendipes* 9233 |
|  |  |  | *Eukiefferiella* 732 |
|  |  |  | *Limnophyes* 1274 |
|  |  |  | *Micropsectra* 4432 |
|  |  |  | *Paratanytarsus* 11 |
|  |  |  | *Polypedilum* 1421 |
|  |  |  | *Pseudosmittia* 26 |
|  |  | Chloropidae 370 | *Gampocsera* 45 |
|  |  |  | *Rhodesiella* 75 |
|  |  | Dolichopodidae 580 | *-* |
|  |  | Drosophilidae 1487 | *Leucophenga* 1200 |
|  |  | Empididae 31569 | *Hilara* 1045 |
|  |  |  | *Rhamphomyia* 24744 |
|  |  | Ephydridae 437 | *Nostima* 39 |
|  |  |  | *Psilopa* 11 |
|  |  |  | *Scatella* 26 |
|  |  |  | *Scatophila* 227 |
|  |  | Hybotidae 21784 | *Anthalia* 17721 |
|  |  |  | *Bicellaria* 220 |
|  |  |  | *Hoplocyrtoma* 32 |
|  |  |  | *Leptopeza* 1644 |
|  |  |  | *Syndyas* 104 |
|  |  | Iteaphila-group 653 | *Iteaphila* 653 |
|  |  | Limoniidae 48 | *Trentepohlia* 26 |
|  |  | Lonchaeidae 56 | *Lonchaea* 38 |
|  |  | Milichiidae 120 | *-* |
|  |  | Muscidae 1866 | *Coenosia* 1866 |
|  |  | Mycetophilidae 1592 | *Anatella* 613 |
|  |  |  | *Boletina* 225 |
|  |  |  | *Docosia* 28 |
|  |  |  | *Novakia* 693 |
|  |  |  | *Phronia* 11 |
|  |  |  | *Sciophila* 10 |
|  |  | Phoridae 36 | *Megaselia* 25 |
|  |  |  | *Phora* 11 |
|  |  | Psychodidae 74531 | *Lutzomyia* 18 |
|  |  |  | *Psychoda* 56995 |
|  |  | Scathophagidae 11 | *Chaetosa* 11 |
|  |  | Scatopsidae 45 | *-* |
|  |  | Sciaridae 1298 | *Baeosciara* 101 |
|  |  |  | *Corynoptera* 81 |
|  |  |  | *Scatopsciara* 22 |
|  |  |  | *Schwenckfeldina* 41 |
|  |  | Sepsidae 105 | *-* |
|  |  | Simuliidae 640 | *Simulium* 640 |
|  |  | Sphaeroceridae 1586 | *Rachispoda* 30 |
|  |  | Tachinidae 114 | *Phytomyptera* 94 |
|  |  |  | *Trichophora* 20 |
|  | Hemiptera 47779 | Cicadellidae 47779 | *Fagocyba* 47673 |
|  |  |  | *Ribautiana* 106 |
|  | Lepidoptera 2401 | Geometridae 72 | *Eariodes* 72 |
|  |  | Notodontidae 13 | *Rifargia* 13 |
|  |  | Tortricidae 2316 | *Epinotia* 1176 |
|  |  |  | *Lepteucosma* 18 |
|  |  |  | *Pseudexentera* 16 |
|  | Psocodea 554 | Ectopsocidae 554 | *Ectopsocus* 554 |
|  |  |  |  |
