# Supplementary material for: DNA metabarcoding allows non-invasive identification of arthropod prey provisioned to nestling Rufous hummingbirds (Selasphorus rufus)
Source: PeerJ. 2019 Mar 5;7:e6596. doi: 10.7717/peerj.6596 (PMC6407503; doi:10.7717/peerj.6596)
Supplement: Table S3 — Numbers after taxon are reads. [file peerj-07-6596-s007.docx]

**Supplemental Table S3.** **Taxonomic breakdown of invertebrates identified from DNA in fecal pellets from five Rufous hummingbird (*Selasphorus rufus*) nests from southern Vancouver Island, British Columbia, Canada, in 2018.** Numbers after taxon are reads.

| **Class (*n*=3)** | **Order (*n*=5)** | **Family (*n*=28)** | **Genus (*n*=35)** |
| --- | --- | --- | --- |
| Arachnida 3540 | Araneae 3540 | Linyphiidae 123 | *Neriene* 123 |
|  |  | Tetragnathidae 1253 | *Metellina* 1253 |
|  |  | Theridiidae 2164 | *Enoplognatha* 2108 |
|  |  |  | *Theridion* 56 |
| Collembola 16 | Entomobryomorpha 16 | Isotomidae 16 | *-* |
| Insecta 190244 | Coleoptera 231 | Scraptiidae 231 | *Anaspis* 231 |
|  | Diptera 189973 | Agromyzidae 1278 | *-* |
|  |  | Bibionidae 1401 | *Dilophus* 1401 |
|  |  | Calliphoridae 86 | *-* |
|  |  | Ceratopogonidae 1661 | *Dasyhelea* 161 |
|  |  |  | *Forcipomyia* 173 |
|  |  | Chironomidae 26824 | *Brillia* 201 |
|  |  |  | *Conchapelopia* 186 |
|  |  |  | *Dicrotendipes* 182 |
|  |  |  | *Glyptotendipes* 24622 |
|  |  |  | *Smittia* 725 |
|  |  | Chloropidae 443 | *Siphonella* 12 |
|  |  | Drosophilidae 505 | *Drosophila* 167 |
|  |  |  | *Leucophenga* 12 |
|  |  |  | *Phortica* 77 |
|  |  | Empididae 23222 | *Rhamphomyia* 12293 |
|  |  | Ephydridae 33 | *Lamproscatella* 12 |
|  |  | Heleomyzidae 26 | *-* |
|  |  | Hybotidae 33464 | *Hoplocyrtoma* 16 |
|  |  |  | *Oedalea* 28623 |
|  |  |  | *Platypalpus* 4627 |
|  |  | Lauxaniidae 1960 | *-* |
|  |  | Limoniidae 106 | *Cladura* 22 |
|  |  |  | *Ormosia* 84 |
|  |  | Milichiidae 53 | *-* |
|  |  | Muscidae 230 | *Atherigona* 174 |
|  |  |  | *Coenosia* 30 |
|  |  |  | *Spilogona* 12 |
|  |  | Mycetophilidae 377 | *Boletina* 13 |
|  |  |  | *Sciophila* 364 |
|  |  | Phoridae 92830 | *Megaselia* 92595 |
|  |  | Psychodidae 441 | *Psychoda* 110 |
|  |  | Simuliidae 4375 | *Simulium* 4375 |
|  |  | Sphaeroceridae 436 | *-* |
|  |  | Syrphidae 47 | *Ceriana* 16 |
|  |  |  | *Psilota* 31 |
|  |  | Tephritidae 53 | *Trypeta* 53 |
|  | Lepidoptera 40 | Tortricidae 40 | *-* |
